# New histological risk grading system for prediction of lymph node metastasis in patients with penile cancer

**DOI:** 10.1007/s00428-024-03916-3

**Published:** 2024-09-09

**Authors:** Luiza Dorofte, Sabina Davidsson, Jessica Carlsson, Gabriella Lillsunde Larsson, Mats G. Karlsson

**Affiliations:** 1 Department of Laboratory Medicine, Faculty of Medicine and Health, Örebro University, Örebro, Sweden; 2 Department of Urology, Faculty of Medicine and Health, Örebro University, Örebro, Sweden; 3 School of Health Sciences, Örebro University, Örebro, Sweden

**Keywords:** Penile cancer, Histological risk grading, Risk groups, Lymph node metastasis

## Abstract

Inguinal lymph node surgery is a standard treatment for penile cancer patients with intermediate or high risk for lymph node metastasis (LNM) according to European Association of Urology (EAU) risk grading. We are proposing a more objective histological prognostic grading system for inguinal LNM in these patients. We assessed worst pattern of invasion, lymphocytic host response, lymphovascular invasion, and perineural invasion in a population-based cohort of 306 penile cancer patients. Patients were classified into low, intermediate, and high risk for inguinal LNM. There was a significant association both between risk groups and pT stage (*p* < 0.001) and between risk groups and LNM. Univariate logistic regression showed 25.43 times higher odds of LNM for patients in the intermediate risk group compared with the low risk group (odds ratio (OR) 25.43; 95% confidence interval (CI): 5.94–108.97) and a 177.13 times higher odds in the high risk group compared to the low risk group (OR 177.13; 95% CI: 40.09–782.51). When comparing our histological risk grading with the EAU grading, we found a higher specificity, of 53.43% (95% CI: 47.84–59.02) versus 15.20% (95% CI: 11.17–19.22), as well as a higher area under the curve (0.86; 95% CI: 0.81–0.89; versus 0.65; 95% CI: 0.58–0.71) with our grading system. While our grading classified 111 patients as low risk, only 31 were considered low risk for LNM according to the EAU risk classification. The new histological risk grading system shows a higher specificity and includes a higher number of patients in the low risk group in whom lymph node surgery could be avoided, reducing morbidity and costs.

## Introduction

Invasive penile squamous cell carcinoma is a rare malignancy, especially in Europe and the United States, with an overall incidence of < 1 per 100,000 men [1, 2]. However, worldwide, the incidence ranges from 0.05 per 100,000 men in North Africa to 1.5 per 100,000 men in South America [1]. In specific parts of Africa, an incidence of 3.7 per 100,000 men has been reported [3]. Although fairly rare, penile tumors are associated with a significant impact on quality of life [4], morbidity, and mortality, with > 13,000 fatal cases worldwide per annum according to a 2020 statistic [1].

Tumor stage and histopathological differentiation predict disease outcome; however, the presence or absence of inguinal lymph node metastasis (LNM) is the most important factor for patient survival [5, 6]. Different clinical and pathological factors, such as tumor grade, tumor stage, lymphovascular invasion (LVI) or perineural invasion (PNI), and histological subtype and growth pattern, have been used for prediction of LNM [7]. At diagnosis, most patients are clinically lymph node-negative, but up to 25% without clinically palpable inguinal lymph nodes have occult micrometastases [7, 8]. Unfortunately, no radiological imaging method is sensitive enough to detect micrometastases [9]. Low risk patients are surveilled, and patients with intermediate and high risk tumors undergo dynamic sentinel node biopsy [10], while the standard treatment for patients with histologically confirmed lymph node-positive disease is radical inguinal lymph node dissection [10]. Because of significant postoperative morbidity, prophylactic radical inguinal lymphadenectomy for all patients with lymph node-negative disease is not recommended. Complication rates of up to 84% have previously been reported in radical inguinal lymphadenectomy [11] and up to 13% in dynamic sentinel node biopsy [12, 13]. Hence, patients are stratified into risk groups (low, intermediate, or high risk) based on histopathological features of the primary tumor [14, 15]. However, currently used risk grading of penile tumors, based on tumor histological grade and stage, in clinical practice has shown low reproducibility [16–18], and no objective method for predicting metastatic disease in patients with penile cancer is yet available.

The aim of the present study in patients with penile cancer was to evaluate a new histological prognostic grading system, inspired by the well-known Brandwein-Gensler risk model for predicting outcome in oral squamous cell carcinoma [19]. The new grading system based on objective histological criteria classifies patients according to risk of developing inguinal LNMs. Better prediction of inguinal lymph node status will lead to improved personalized treatment planning.

## Materials and methods

We retrieved formalin-fixed, paraffin-embedded tumor tissue from 349 patients surgically treated for penile cancer at Örebro University Hospital, Örebro, Sweden, between 2009 and 2018. The material is population-based, deriving from the major part of the Swedish health care system. We microscopically examined slides with hematoxilin and eosin-stained tissue from all primary invasive tumors. All the cases were squamous cell carcinoma and were classified into histological subtypes and graded according to histological criteria recommended by the World Health Organization (WHO) for classification of tumors [20]. In accordance with the treatment protocol for penile cancer based on national standards and European Association of Urology (EAU) recommendations from 2014 [15], tumors with a low risk of LNM (pT1G1) had been followed with active monitoring of lymph node status. In patients with tumors with intermediate risk (pT1G2) and high risk (> pT1G2) for LNM, a dynamic sentinel node biopsy had been performed [10, 15]. We excluded patients with intermediate or high risk for LNM according to the EAU risk grading system, in whom inguinal lymph node surgery had not been performed (*n* = 39) because of poor patient health. In addition, four patients were excluded because of limited tumor material and missing pT stage (*n* = 2) or because of diagnosis of a pTa tumor (*n* = 2). The remaining 306 cases, 102 of whom had presented with LNM at diagnosis, were included in the analysis (Table 1).

**Table 1:** Clinical characteristics of the included patients with penile cancer at diagnosis

	*N* = 306
Age at diagnosis, mean (sd)	67.8 (10.6)
pT^*^ stage, n (%)	
pT1	109 (35.6)
pT1a	82 (26.8)
pT1b	27 (8.8)
pT2	126 (41.2)
pT3	71 (23.2)
Lymph node metastases, n (%)	
Yes	102 (33.3)
No	204 (66.7)

*sd*, standard deviation

^*^T1: Tumour invades subepithelial connective tissue; T1a: G1-G2 tumour with invasion in subepithelial connective tissue without lymphovascular invasion or perineural invasion; T1b: Tumor with invasion in subepithelial connective tissue with lymphovascular invasion or perineural invasion or G3 tumor

T2 Tumour invades corpus spongiosum

T3 Tumour invades corpus cavernosum

**Fig. 1:**
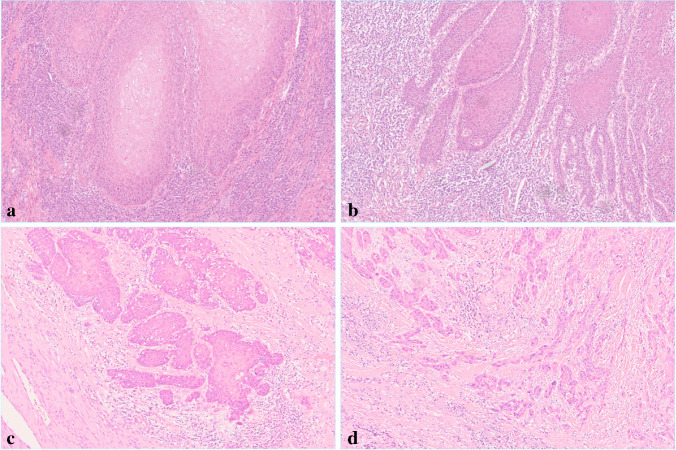
Worst pattern of invasion (WPOI): hematoxylin–eosin stain, magnification × 100. (**a**) type 1, (**b**) type 2, (**c**) type 3, (**d**) type 4

**Fig. 2:**
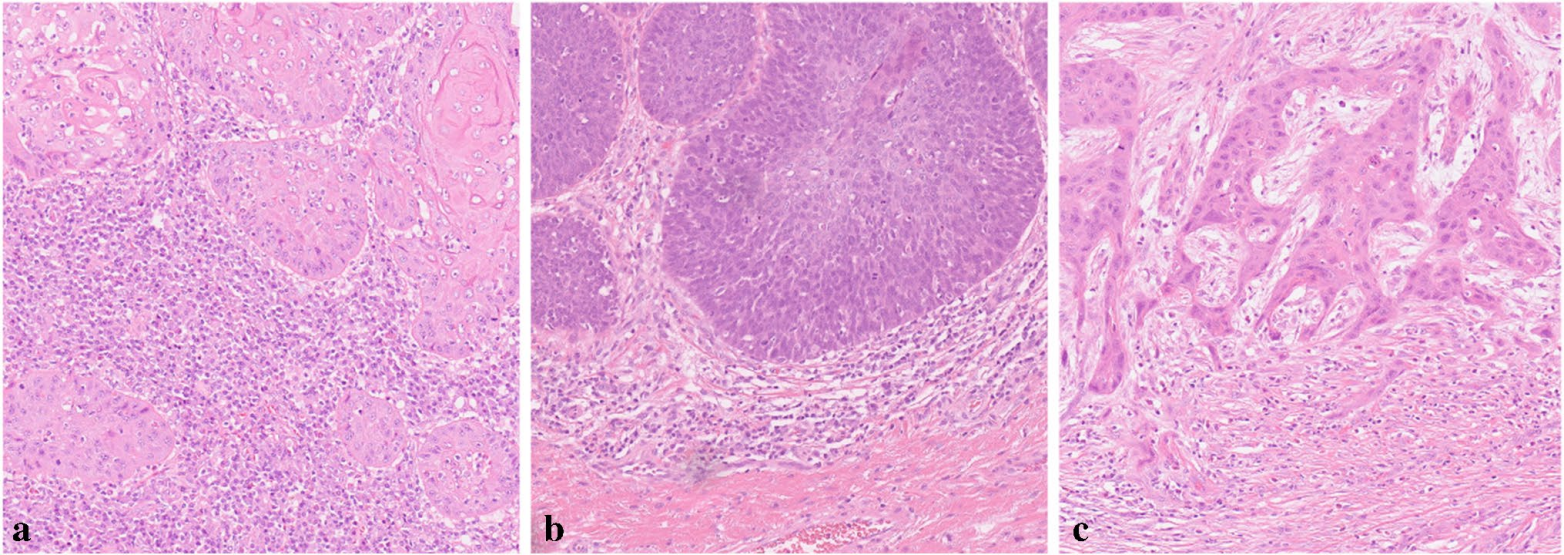
Lymphocytic host response: hematoxylin–eosin stain, magnification × 200. (**a**) type 1, (**b**) type 2, (**c**) type 3

A pathologist with experience in uropathology (L.D.) reviewed all slides and evaluated the following four histological parameters: worst pattern of invasion (WPOI) (Fig. 1), lymphocytic host response (LHR) (Fig. 2), LVI, and PNI, as defined in Table 2. The status of the lymph nodes was unknown during the data collection. The patients were classified into low, intermediate, and high risk groups based on the summated points (Table 2). A total point score of 0 = low risk tumor; 1 or 2 = intermediate risk tumor; and ≥ 3 = high risk tumor was calculated.

**Table 2:** Histopathological parameters and point assignment in the proposed histological risk grading system

Histopathological parameter	Type and definition	Point assignment
WPOI	Type 1 – Pushing border	0
	Type 2 – Finger-like growth	0
	Type 3 – Large separate islands, > 15 cells per island	0
	Type 4 – Small tumor islands, ≤ 15 cells per island	+ 1
LHR	Type 1 – Strong peritumoral host response, with lymphoid nodules in each 4 × field	0
	Type 2 – Intermediate peritumoral host response, with lymphoid nodules in some but not all 4 × fields	+ 1
	Type 3 – Little or no host response, with no lymphoid nodules	+ 2
LVI	Absent	0
	Present	+ 1
PNI	Absent	0
	Present	+ 1

*WPOI*, worst pattern of invasion; *LHR*, lymphocytic host response; *LVI*, lympho-vascular invasion; *PNI*, perineural invasion

### Ethical approval

The study was approved by the Swedish Ethical Review Authority (2019–01923) (Biobank approval 18RS57). The study was performed in accordance with the Declaration of Helsinki.

### Statistics

Categorical data was described using absolute and relative frequencies. Chi-square tests or Fisher’s exact tests were performed to test for association between lymph node status and risk groups. The sensitivity and specificity of the risk score were assessed by comparing the low risk group with the intermediate and high risk group. Logistic regression models were used to estimate odds ratios (ORs) and 95% confidence intervals (CIs) for the association between lymph node status and risk groups. To evaluate the discriminating capacity of the risk group system, a receiver operating characteristics (ROC) analysis was performed with calculation of the area under the curve (AUC) for LNM. Cohen’s kappa statistics were calculated to investigate the agreement between the two risk scores, as well as sensitivity, specificity, positive predictive value (PPV), and negative predictive value (NPV). Statistical significance was set at *p* < 0.05. All analyses were performed using IBM SPSS Statistics, version 27 (IBM Corp., Armonk, NY, USA), and STATA (StataCorp, College Station, TX, USA).

## Results

The patients’ clinical characteristics are summarized in Table 1. Of the 306 patients included in the study, the majority were diagnosed with a pT1 or pT2 tumor (35.6% and 41.2%, respectively), and 33.3% of the patients had LNMs at diagnosis. Histopathological characteristics as well as risk classification of the tumors are summarized in Tables 3 and 4.

**Table 3:** Histopathological characteristics of patients with penile cancer, according to the proposed histological risk grading system

Histopathological characteristics	*N* = 306
WPOI, n (%)	
Type 1	8 (2.6)
Type 2	53 (17.3)
Type 3	135 (44.1)
Type 4	110 (36.0)
LHR, n (%)	
Type 1	176 (57.5)
Type 2	92 (30.0)
Type 3	38 (12.5)
LVI, n (%)	
Yes	112 (36.6)
No	194 (63.4)
PNI, n (%)	
Yes	85 (27.8)
No	221 (72.2)

*WPOI*, worst pattern of invasion; *LHR*, lymphocytic host response; *LVI*, lympho-vascular invasion; *PNI*, perineural invasion

**Table 4:** Number of patients in each risk group, according to the proposed histological risk grading system and the European Association of Urology (EAU) grading system

Risk group	Low risk	Intermediate	High risk
Proposed histological risk grading	111	110	85
EAU risk grading	31	55	220

### Risk group stratification

There was a significant association between risk groups in the proposed histological risk grading system and pT stage (*p* < 0.001). The majority of the patients in the low risk group had pT1 (56.7%) or pT2 tumors (35.1%). In the intermediate and high risk groups, the majority of patients had pT2 tumors (43.4% and 46.1%, respectively); 31.1% of patients in the intermediate risk group had pT1 tumors, and 39.3% in the high risk group had pT3 tumors (Table 5).

**Table 5:** Risk group characteristics in the proposed histological risk grading system concerning pT stage and lymph node metastases at diagnosis

	pT stage			Lymph node metastases		
	pT1	pT2	pT3	Yes	No	Total
Low risk	63	39	9	2	109	111
Intermediate	33	46	27	35	71	106
High risk	13	41	35	65	24	89
Total	109	126	71	102	204	306

A significant association was also found between risk groups in the proposed histological risk grading system and LNM (*p* < 0.001). The majority of patients (98.2%) in the low risk group had benign inguinal lymph nodes, while 31.8% of patients in the intermediate risk group and 76.5% of patients in the high risk group had LNM (Table 5).

According to the EAU risk classification, none of the patients in the low risk group, compared with 18.2% in the intermediate risk group and 41.8% in the high risk group, had LNM.

Univariate logistic regression showed that the odds of LNM were 25.43 times higher for patients in the intermediate risk group compared with the low risk group (OR 25.43; 95% CI: 5.94–108.97). They were 177.13 times higher in the high risk group compared to the low risk group (OR 177.13; 95% CI: 40.09–782.51). When adjusting for pT stage and age, increased odds of LNM for patients in the intermediate and high risk groups compared to patients in the low risk group remained statistically significant (Table 6).

**Table 6:** Logistic regression analysis of risk groups, according to the proposed histological risk grading system, and lymph node metastases

	Odds ratio (95% CI)		
	Crude model	Adjusted model^a^	Adjusted model^b^
Low risk	1 (reference)	1 (reference)	1 (reference)
Intermediate risk	25.43 (5.94–108.97)	20.48 (4.70–89.17)	24.22 (5.59–105.07)
High risk	177.13 (40.09–782.51)	135.33 (29.42–622.49)	161.39 (35.21–739.69)

^a^Adjusted for pT-stage (pT1, pT2, and pT3)

^b^Adjusted for pT-stage and age at diagnosis

When investigating the results of EAU risk grading, univariate logistic regression showed no significant difference in the odds of LNM in the low risk group compared to the high risk group (OR 0.00; 95% CI: 0.00–infinity). However, a significant difference in the odds of LNM between the intermediate and high risk group was seen (OR 0.32; 95% CI: 0.15–0.66).

A regression analysis using only the total score showed a nice dose–response relationship, where the higher the score, the higher the odds for metastasis (data not shown).

### Subanalyses of pT stage in the proposed histological risk grading system

Logistic regression analyses of different pT stages were performed separately. No statistically significantly increased odds of LNM were found for patients in the intermediate or high risk group compared to the low risk group regarding either pT2 or pT3 tumors (data not shown). However, increased odds of LNM were seen for both intermediate and high risk group patients compared to low risk group patients regarding pT1 tumors (OR 11.73; 95% CI: 2.4–57.3, and OR 122.0; 95% CI: 15.03–990.26, respectively). Adjusting for age did not change this association (Table 7).

**Table 7:** Logistic regression analysis of risk groups in the proposed histological risk grading, regarding lymph node metastases in patients with pT1 tumors

	Odds ratio (95% CI)	
	Crude model	Adjusted model^a^
Low risk	1 (reference)	1 (reference)
Intermediate risk	11.73 (2.4–57.3)	10.99 (2.24–54.01)
High risk	122.0 (15.03–990.26)	124.84 (14.89–1046.49)

*CI*, confidence interval. ^a^Adjusted for age

### Comparison of proposed histological risk grading and EAU risk grading

There was no agreement between the risk groups identified by the proposed risk grading system and those identified using the EAU risk grading system (kappa = 0.16; *p* < 0.001).

The sensitivity and specificity of the risk score in the proposed histological risk grading system and the EAU risk grading system were calculated comparing low risk with intermediate and high risk group patients (Table 8). When performing ROC curve analysis for predicting LNM, the risk score in the proposed histological risk grading had a higher AUC (0.86; 95% CI: 0.81–0.89) than did the risk score in the EAU risk grading (0.65; 95% CI: 0.58–0.71). Furthermore, the PPV was higher in the proposed histological risk grading compared to the EAU risk grading system (51.28% versus 37.09%). However, the EAU risk grading system had a higher NPV compared to the proposed histological risk grading system (100% versus 98.20%) (Table 8).

**Table 8:** Sensitivity and specificity of the risk score in the proposed histological risk grading and the EAU risk grading systems. Data is given as percentages and 95% confidence intervals

	PPV	NPV	Sensitivity	Specificity	AUC
Proposed histological risk grading	51.28% (45.68–56.88)	98.20% (96.71–99.69)	98.04% (96.49–99.59)	53.43% (47.84–59.02)	0.86 (0.82–0.9)
EAU risk grading	37.09% (31.68–42.50)	100.0% (100.0–100.0)	100.0% (100.0–100.0)	15.2% (11.17–19.22)	0.65 (0.58–0.71)

*AUC*, area under the curve; *NPV*, negative predictive value; *PPV*, positive predictive value

## Discussion

Finding a reliable method for correct prediction of LNM in patients with invasive penile cancer is a major challenge in penile cancer care and has been the aim of some previous studies [21–23]. In clinical practice, patients at risk for LNM who can benefit from inguinal lymph node surgery are identified based on histological grade and tumor stage [8, 15]. Patients with clinically benign lymph nodes and low risk tumors, such as pTa and pT1G1, are at low risk for LNM and can benefit from organ-sparing surgery and lymph node surveillance. Patients with tumors in the intermediate risk group (pT1G2) and high risk group (pT1G3, pT2, pT3, and pT4) undergo dynamic sentinel node biopsy as part of standard treatment [2, 10].

Histological grade is a well-known prognostic factor and is used as a criterion for risk grading in a variety of tumors in different locations. However, tumor grading is a subjective assessment, with low agreement between different pathologists [16–18, 24]. Despite low reproducibility, histological grade represents one of two criteria used in the EAU risk grading system to identify penile cancer patients who can benefit from lymph node surgery.

The first known author who, in 1920, proposed a system for stratifying patients with cancer was Broders, who published research on carcinoma of the lip [25]. Since the 1920s, multiple grading systems have been developed for different types of tumors in various anatomical locations to predict risk of metastasis, local recurrence, and disease progression. Multivariable histological grading systems have shown a better predictive value than have single prognostic variables [19, 26, 27]. In a review article from 2015, Sanchez et al. [28] included data from multiple studies on prognostic factors in penile cancer and developed histological risk groups based on tumor grade and histological subtype. Several other histopathological risk assessment systems have been proposed for predicting LNM in patients with penile cancer. For example, Hungerhuber et al. [29] used risk grading based on tumor grade and stage, and Sali et al. [30] proposed a risk grading system based on tumor grade stage and pattern of infiltration. All these risk grading systems including the EAU guidelines include tumor grade and/or histological subtype among the criteria. In our previous international study, we showed that the assessment of both tumor grade and histological subtype in penile cancer is subjective, with low reproducibility [18]. Therefore, there is a need for further efforts to develop a grading system with objective parameters and prognostic performance.

In the present study, we propose a new histological risk grading system that does not include tumor histological grade among the variables, the aim being to create a more reproducible system based on objective histological parameters. A more objective assessment method for the prediction of inguinal lymph node status could be a step forward in correctly selecting penile cancer patients who will benefit from inguinal lymph node surgery.

Our risk grading is partly inspired by a histological risk assessment method with a high predictive value, the Brandwein-Gensler risk model used in patients with oral squamous cell carcinoma [19]. This grading method is based on WPOI, PNI, and LHR as well as on earlier histological grading models most often used in squamous cell carcinoma of the head and neck [31–33]. We chose to modify this grading system and include an additional parameter, lymphovascular invasion (LVI).

Pattern of invasion at the tumor interface has shown a high predictive value in different studies including studies of penile cancer patients [7, 33, 34]. Brandwein-Gensler et al., in their study on oral cancer patients, refined the term by validating the use of areas with WPOI [19]. In our proposed histological risk grading system, we use only four types of pattern of invasion, as defined by Bryne et al. [26, 31] (Table 2). Perineural invasion has also been linked to an increased risk of LNM, especially for high grade tumors [35, 36]. The association between LHR and disease progression has been reported in different studies that have shown that the level of peritumoral inflammatory cell infiltrate has been associated with tumor pT stage and disease outcome in patients with penile cancer [37–39]. As well as the prognostic value, LHR response may even play a role in immune-based therapies [40].

Besides the three parameters (WPOI, PNI and LHR) described above, we have included LVI, which has shown a good prognostic value in predicting LNM in earlier studies on penile cancer [7, 41, 42]. Lymphovascular invasion has even been included as a criterion for staging of penile tumors. Thus, penile cancer stage pT1 has been divided into pT1a (G1–G2 without vascular invasion) and pT1b (G3 and/or vascular invasion), according to the latest tumor node metastasis (TNM) classification [43].

We chose to not include histological subtype and human papillomavirus (HPV) tumor status among the variables as, in a previous study, we showed that the assessment of histological tumor subtype is subjective, with low agreement between pathologists. In many low-income countries, HPV analysis or p16 stain is still unavailable.

Our results show that presence or absence of metastasis in the inguinal lymph nodes was significantly related to the total number of points and classification into risk groups. Important differences were observed between patients in different risk groups. Metastases were almost non-existent in the low risk group, while we found a significant correlation between the intermediate and high risk group and the presence of nodal metastasis.

A comparison between our proposed histological risk grading and EAU risk grading shows that a much higher number of patients were included in the low risk group in our grading. Even though this risk group included patients with pT1, pT2, and pT3 penile tumors, our grading still had a very high specificity. If we apply our risk grading only to patients with pT1 tumors, we get a specificity of 100% (data not shown). While part of pT1 tumors as well as all stage pT2 and pT3 tumors were included in the intermediate and high risk group, according to EAU grading, many of these patients, according to our model, had a low risk for metastases and, therefore, lymph node surgery was not necessary. At the same time, the proposed histological risk grading system has shown a higher specificity and AUC than the currently used EAU grading system and is able to better divide patients into appropriate risk groups regarding LNM.

The new histological risk grading system that we are proposing showed a better PPV than the EAU grading and is therefore able to better identify patients who could benefit from inguinal lymph node surgery rather than surveillance. Thus, fewer patients would need inguinal surgery, with reduced costs and risk for complications. However, the NPVs and PPVs in this study should be interpreted with caution, because the low prevalence of LNM can have an impact on both measurements. In future studies, if our results are reproduced in other high volume centers representing different demographic and geographic groups, this risk grading system may become a really useful prediction method that could be used in clinical practice when choosing the best treatment management for patients with penile cancer.

Our study has some strengths and limitations. The proposed risk grading system could be used by less experienced pathologists as it is based on more objective criteria, such as PNI, LVI, and growth patterns. While, from a statistical point of view, the total number of patients included in this study is not that high, few other studies are based on such a considerable cohort of patients with penile cancer taking into consideration the rarity of this type of cancer. Our results show high odds of metastatic disease for patients in the high risk group; however, the CIs are wide and the OR for metastatic disease should be interpreted with caution. Further retrospective studies in other centers specialized in treatment of penile cancer are needed to validate the proposed risk grading system. Our next step is a validation study in collaboration with other international centra, specialized in diagnosis of penile cancer. We are planning to involve pathologists with different experience level to prove the objectivity and applicability of the proposed grading system. If our results are reproduced by future studies, then the method could be used in clinical settings in the triage of penile cancer patients and possibly other types of cancer patients. Machine learning analysis could be used in future studies to potentially enhance the predictive accuracy of the proposed histological risk grading system.

## Conclusions

A more accurate risk grading system for penile tumors is still needed to improve the clinical management of penile cancer patients. Our proposed histological risk grading system better predicts the lymph node status of patients with penile cancer compared to other grading system in use. While patients with intermediate and high risk for inguinal LNM are still identified, more patients could be offered surveillance without surgical intervention. However, further validation studies are needed.

## Data Availability

The datasets generated during and/or analysed during the current study are available from the corresponding author on reasonable request.
