# Correction to: New histological risk grading system for prediction of lymph node metastasis in patients with penile cancer

**DOI:** 10.1007/s00428-025-04112-7

**Published:** 2025-04-29

**Authors:** Luiza Dorofte, Sabina Davidsson, Jessica Carlsson, Gabriella Lillsunde Larsson, Mats G. Karlsson

**Affiliations:** 1 Department of Laboratory Medicine, Faculty of Medicine and Health, Örebro University, Örebro, Sweden; 2 Department of Urology, Faculty of Medicine and Health, Örebro University, Örebro, Sweden; 3 School of Health Sciences, Örebro University, Örebro, Sweden

**Correction to: Virchows Archiv**.

10.1007/s00428-024-03916-3.

In the published paper, the statistical terms of Table 8 headers are not in the right order. This is currently presented as: Sensitivity, Specificity, PPV and NPV. The correct presentation should be: PPV, NPV, Sensitivity and Specificity.

Based on these adjustments, minor corrections in the abstract and results as well as in the discussion sections were also needed to be corrected to correspond to the changes.

The original article has been corrected.

